# Retinoic acid receptor-α signalling antagonizes both intracellular and extracellular amyloid-β production and prevents neuronal cell death caused by amyloid-β

**DOI:** 10.1111/j.1460-9568.2010.07426.x

**Published:** 2010-10

**Authors:** C I Jarvis, M B Goncalves, E Clarke, M Dogruel, S B Kalindjian, S A Thomas, M Maden, J P T Corcoran

**Affiliations:** 1 The Wolfson Centre For Age-Related Diseases, King’s College LondonGuy’s Campus, London SE1 1UL, UK; 2 Pharmaceutical Science Division, King’s College LondonUK; 3 MRC Centre for Developmental Neurobiology, King’s College LondonUK

**Keywords:** ADAM10, Alzheimer's disease, neuronal survival, RARα, retinoic acid, small molecule

## Abstract

Alzheimer’s disease (AD) is characterized by amyloid-β (Aβ) deposition in the brain, neuronal cell loss and cognitive decline. We show here that retinoic acid receptor (RAR)α signalling *in vitro* can prevent both intracellular and extracellular Aβ accumulation. RARα signalling increases the expression of a disintegrin and metalloprotease 10, an α-secretase that processes the amyloid precursor protein into the non-amyloidic pathway, thus reducing Aβ production. We also show that RARα agonists are neuroprotective, as they prevent Aβ-induced neuronal cell death in cortical cultures. If RARα agonists are given to the Tg2576 mouse, the normal Aβ production in their brains is suppressed. In contrast, neither RARβ nor γ-agonists affect Aβ production or Aβ-mediated neuronal cell death. Therefore, RARα agonists have therapeutic potential for the treatment of AD.

## Introduction

The pathological hallmarks of Alzheimer’s disease (AD) are the presence of senile plaques containing amyloid-β (Aβ) peptide and the formation of neuronal tangles in the cerebral cortex. In addition, intraneuronal formation of Aβ has been shown to be involved in AD (LaFerla *et al.*, 2007). Aβ is derived from the amyloid precursor protein (APP), which can also be processed into a non-amyloidic pathway (Thinakaran & Koo, 2008). In the amyloidic pathway, APP is cleaved by β-secretase and then γ-secretase to give Aβ (Thinakaran & Koo, 2008). In the non-amyloidic pathway, α-secretase cleaves the APP within the Aβ sequence, liberating secretory APP (sAPP)α; cleavage by γ-secretase then liberates the non-toxic peptide p3 (Thinakaran & Koo, 2008).

In AD, there are genetic linkages to the disease that are close to genes involved in the retinoid signalling pathway (Goodman & Pardee, 2003). This pathway is mediated by retinoic acid (RA) receptors (RARs) and retinoid X receptors (RXRs), both of which have three types, α, β and γ, and various isoforms (Bastien & Rochette-Egly, 2004). Transcription occurs when the small lipophilic molecule RA binds to an RAR–RXR heterodimer, which then binds to RA response elements located in the regulatory regions of target genes (Bastien & Rochette-Egly, 2004).

RA is derived from vitamin A, and we have shown that a deficiency in rats leads to Aβ deposits in the brain vasculature and neuronal cell death (Corcoran *et al.*, 2004). This was correlated with the lack of RARα signalling, as this receptor is downregulated in vitamin A-deficient rats, and the same receptor deficit is found in the cortices in pathology samples of AD patients (Corcoran *et al.*, 2004). Recent work has also shown that all-trans RA (atRA) applied intraperitoneally in a mouse model of AD results in a decrease in Aβ production (Ding *et al.*, 2008), but the specific RAR involved in this process was not identified. If retinoids are to be used as therapeutic agents for AD, it is important to identify the specific RAR(s) involved in this process in order to discover relevant drug targets, thus overcoming deficiencies in atRA, which can activate all of the RARs, with potential toxic effects. Although the retinoid agonists used here are not approved as drugs, they can act as starting points for effective therapeutics.

In this study, we show *in vitro* that both intracellular and extracellular Aβ production and the neuronal cell death associated with Aβ can be prevented by RARα signalling, and that there is induction of a disintegrin and metalloproteinase (ADAM)10, which has been shown to act as an α-secretase (Lammich *et al.*, 1999; Endres *et al.*, 2005), and whose modulation may be an opportunity for the treatment of AD (Endres & Fahrenholz, 2010). In addition, we show that an RARα agonist can cross the blood–brain barrier (BBB) and that RARα agonists administered to Tg2576 mice either intraperitoneally or orally can prevent Aβ production via an upregulation of ADAM10.

## Materials and methods

### Neuronal cultures

Cortical neurons were isolated from embryonic day 15 mice (NIH Swiss; Harlen, Bicester, UK). The brains were removed from the embryos, and washed three times in phosphate-buffered saline (PBS)–1.5% glucose. Cortices with their meninges removed were then triturated through a 21G needle in the presence of PBS–1.5% glucose. The dissociated cells were then left for 5 min on ice to allow debris to settle. The supernatant was transferred to a 15-mL falcon tube and spun for 5 min at 134 ***g***. Tissue culture plates (Nunc, Invitrogen, Paisley, UK) or 13-mm^2^ glass coverslips in 24-well plates (Nunc, Invitrogen) for immunohistochemistry were precoated with 10 μg/mL poly(d-lysine) (Sigma Aldrich, Dorset, UK). Cells were plated at a density of 2 × 10^6^ per well in six-well plates for protein and RNA isolation, 0.5 × 10^6^ per well in 24-well plates for immunohistochemistry and enzyme-linked immunosorbent assays (ELISAs), and 0.5 × 10^5^ cells per well in 96-well plates for neuronal cell survival assays.

The neurons were grown in Neurobasal medium (Invitrogen, Paisley, UK), supplemented with B-27 (Invitrogen, Paisley, UK), 2 mm glutamine, 1.5% glucose, 100 μg/mL streptomycin and 60 μg/mL penicillin (Invitrogen) at 37°C in a humidified atmosphere of 95% air and 5% CO_2_. Cultures were used after 7 days *in vitro*, and were composed of > 98% neurons as judged by β-tubulin III staining. All of the cultures used were similar in appearance at the time of treatment. Dexamethasone (Sigma Aldrich, Dorset, UK) and the retinoids were used in × 1000 stock concentrations in dimethylsulphoxide. The retinoids used have been previously described (Bernard *et al.*, 1992; Corcoran *et al.*, 2000; Goncalves *et al.*, 2009). They were: atRA (Sigma Aldrich, Dorset, UK); two RARα selective agonists (AM 580, referred to here as RARαa and BMS 194753, referred to here as RARαb); a RARα selective agonist (CD2019) and a RARγ selective agonist (CD437). All of the retinoid agonists were synthesized by Sygnature Chemical Services (Nottingham, UK) except for BMS 194753, which was provided as a gift by C. Zusi of BMS. Culture conditions were three wells per treatment carried out three times.

### Neuronal survival

Aβ1–40 and Aβ1–42 (Californian Peptide Research, Napa, CA, USA) were made up at × 1000 concentration in dimethylsulphoxide. Neurons were cultured for 7 days in serum-free medium and then for 3 days with 10 μm human Aβ and either 0.01, 0.1 or 1 μm retinoid agonist. Cell survival was assessed with a 3-(4,5-dimethylthiazol-2-yl)-2,5-diphenyltetrazolium bromide (MTT) assay (Promega, Southampton, UK), according to the manufacturer’s instructions. Alternatively, at the end of the treatment period, the medium was changed to PBS containing 50 μm propidium iodide (Sigma Aldrich) and 250 μm Hoechst (Sigma Aldrich), and after 15 min the number of viable cells was counted on an IN Cell Analyser 1000 (GE Healthcare, Little Chalfont, Bucks, UK).

### ELISAs

Aβ1–40 and Aβ1–42 were measured with sandwich ELISA kits according to the manufacturer’s instructions [Wako Chemicals (GmbH, Neuss, Germany) for mouse Aβ, and Invitrogen for human Aβ]. Secreted Aβ from the *in vitro* assays was measured by the direct addition of the cell medium to the ELISA plates in the presence of protease inhibitors (Calbiochem, Merck Chemicals, Nottingham, UK). For measurement of intracellular Aβ from the *in vitro* assays, the cells were washed twice in PBS, and the Aβ was extracted with 5 m guanidine hydrochloride/50 mm Tris–HCl (pH 8.0). This was mixed for 3–4 h at room temperature, and then diluted 1 : 20 in PBS containing 5% bovine serum albumin and 0.03% Tween-20 and centrifuged at 16 000 ***g*** for 20 min at 4°C. The supernatant was then added to the ELISA plates in the presence of protease inhibitors. For analysis of Aβ in Tg2576 mice, animals were perfused with saline and brains were dissected out. Cortices were then snap frozen and stored at −80°C until use. For analysis of Aβ, the cortices were homogenized in 10 volumes of ice-cold 5 m guanidine hydrochloride/50 mm Tris–HCl (pH 8.0), and the samples were processed as above. Aβ readings were normalized to protein concentrations of the cells or brain tissue used, in order to eliminate variability in cell numbers. The assays were performed in triplicate for each animal.

### RT and real-time PCR

RNA was isolated as previously described (Corcoran *et al.*, 2000), and cDNA synthesis was carried out with an Amersham kit, according to the manufacturer’s instructions. The following mouse primers were used: ADAM10, forward, aaagaccctacaaatcctttcc; ADAM10, reverse, gcttttctcacatattccccc (product length 192 bp); β-site of amyloid precursor protein-cleaving enzyme (BACE1), forward, atcagtccttccgcatcac; BACE1, reverse, gcaaagccaattcgctttc (product length 187 bp); mouse γ-secretase, forward, ctcatctacacgcccttcac; mouse γ-secretase, reverse, catcagggaggacatgatcag (product length 180 bp); and glyceraldehyde-3-phosphate dehydrogenase (GAPDH), forward, cgtagacaaaatggtgaaggt; GAPDH, reverse, gactccacgacatactcagc (product length 297 bp). Real-time PCR was performed using an SYBRGreen kit (Roche Products, Nottingham, UK), and a Roche light cycler, 250 ng of cDNA and the specific primer pairs (0.5 μm each primer). The quantitative PCR was performed as follows: heating to 95°C for 5 min, followed by 45 PCR cycles (one cycle contained the following steps: 5 s at 95°C, 5 s at 55°C, and 15 s at 72°C). The specificity of each primer pair was confirmed by melting curve analysis and agarose gel electrophoresis. The relative quantity of mRNA was calculated from a GAPDH standard curve.

### Antibodies

The following antibodies were used: rabbit α-ADAM10 (1 : 100; Millipore, Livingston, UK), mouse anti-APP (1 : 200, 22C11; Millipore), mouse anti-Aβ (1 : 5000, 6E10; Covance, Emeryville, CA, USA) and mouse anti-βIII tubulin (1 : 1000; Promega). Secondary antibodies were AlexaFluor 594 (1 : 1000; Molecular Probes, Invitrogen, Paisley, UK) and AlexaFluor 680 (1 : 5000; Molecular Probes).

### Immunohistochemistry and image analysis

Immunohistochemistry was carried out as previously described (Goncalves *et al.*, 2005). Neuronal cultures were washed with PBS for 1 min. They were then fixed in 4% paraformaldehyde for 30 min, and washed three times for 5 min each in PBS–0.02% Tween. They were incubated in primary antibody in PBS–0.02% Tween at 4°C overnight. Primary antibody was removed by washing three times for 5 min each in PBS–0.02% Tween. They were incubated in the secondary antibody for 1 h at room temperature in PBS–0.02% Tween, and then washed in PBS three times for 5 min each. The first wash contained the nuclear stain 4′,6-diamidino-2-phenylindole dihydrochloride (DAPI) (1 μg/mL; Sigma Aldrich). The coverslips were then mounted with FluroSave reagent (Merck, Hoddeson, Hertfordshire, UK). Incubation of neurons with secondary antibodies in the absence of primary antibodies produced a very weak diffuse staining of cell bodies that did not overlap with the primary antibody-specific staining (data not shown). Multichannel fluorescence (DAPI–fluorescein isothiocyanate–Texas Red filter set) images were captured with axiovision (release 4.6) imaging software and a Plan-Apochromat ×20 0.75 NA objective fitted to an ApoTome workstation (Axioplan 2 imaging microscope with Apotome slider module, motorized focus, and an AxioCam MRm cooled monochrome digital camera set at 1388 × 1040 pixel resolution) (Carl Zeiss). Channels were imaged sequentially to eliminate bleed-through, and multichannel image overlays were obtained with adobe photoshop 7.0 (Adobe Systems, San Jose, CA, USA).

### Western blotting

Protein was isolated from the neuronal cultures in a lysis solution containing 50 mm Tris–HCl (pH 7.6), 1% NP-40 (Sigma Aldrich), 150 mm NaCl and 2 mm EDTA, with 1 mm phenylmethylsulfonyl fluoride, in the presence of a protease inhibitor mixture (Sigma Aldrich). Cell lysates were centrifuged for 10 min at 9 000 ***g*** Extracellular protein was isolated from the treated Tg2576 mice as previously described (Lesne *et al.*, 2006). The protein concentration in the supernatants was determined with the BCA Protein Assay (Pierce, Rockford, IL, USA). Ten micrograms or 2.5 μg of protein was loaded onto 8% SDS-PAGE gels. Wet blotting was performed, and the blots were probed with 22C11 to detect full-length APP or 6E10 to detect sAPPα. The membranes were then incubated with the secondary antibody and visualized with an Odyssey infrared scanning system. For a loading control, the blots were probed with mouse anti-βIII tubulin and developed as above. Signal density was calculated as the ratio of signal intensity to β-III tubulin.

### Animal treatments

All procedures were performed in accordance with the Animal Scientific Procedures Act 1986 (UK). Mice (129S2/SvHsd) were purchased from Harlen, and Tg2576 mice on a 129S6 background were purchased from Taconic Farms (Germantown, NY, USA). Mice were maintained on a 12-h light/dark cycle at 20–22°C and given food and water *ad libitum*. Mice were injected intraperitoneally with 1 mg/kg retinoids three times a week from 3 to 7 months of age, or fed retinoids at 3.6 mg/kg daily from 3 to 7 months of age. These doses were based on previous work (Hind & Maden, 2004; Stinchcombe & Maden, 2008). For intraperitoneal studies, *n* = 5, and for feeding studies, *n* = 3.

### *In situ* brain perfusion technique

The perfusion method was used as previously described (Sanderson *et al.*, 2008). Adult male 129S2/SvHsd mice (∼25 g) were anaesthetized [intraperitoneal medetomidine hydrochloride (2 mg/kg), ketamine (150 mg/kg) and heparin (100 U)]. The flow rate was 5 mL/min for 10 min. AM 580 (*M*_r_ 355.2) was custom radiolabelled with tritium (54.8 Ci/mmol, radiochemical purity 99.9%; Moravek, Brea, CA, USA), and [^14^C]sucrose (*M*_r_ 342, 498 mCi/mmol, radiochemical purity ≥ 98%; Moravek). [^3^H]AM 580 (4.6 nm) and [^14^C]sucrose (1.5 μm) were present in the artificial plasma. The artificial plasma consisted of a modified Krebs–Henseleit mammalian Ringer solution with the following constituents: 117 mm NaCl, 4.7 mm KCl, 2.5 mm CaCl_2_, 1.2 mm MgSO_4_, 24.8 mm NaHCO_3_, 1.2 mm KH_2_PO_4_, 10 mm glucose, and 1 g/L bovine serum albumin. After perfusion, a cisterna magna cerebrospinal fluid (CSF) sample was taken, the animal was decapitated and the brain was removed. Samples of the frontal cortex, occipital cortex, caudate putamen, hippocampus, amygdala, hypothalamus, thalamus, pons, cerebellum and fourth ventricle choroid plexus were taken. All of the brain matter remaining after these samples had been taken was subjected to capillary depletion analysis (Sanderson *et al.*, 2008). In brief, a brain homogenate was prepared, using a buffer and a dextran solution. The final dextran concentration was 13%. Centrifugation of this homogenate (5,400 ***g*** for 15 min at 4°C) produced an endothelial cell-enriched pellet and a brain parenchyma-containing supernatant. Capillary depletion (including brain homogenate, supernatant and pellet), brain region, circumventricular organ, CSF and plasma samples were solubilized with 3.5 mL of Solvable (Perkin Elmer, Walthan, MA, USA; 0.5 mL). Lumasafe scintillation fluid (Perkin Elmer) was then added. Sample radioactivity was quantified (Packard Tri-Carb 2900TR counter). Tissue radioactivity (d.p.m./g) was expressed as a percentage of that in plasma (d.p.m./mL) and termed *R*_Tissue_ (mL/100 g). Where stated, the *R*_Tissue_ for AM 580 has been corrected for vascular/extracellular space by subtraction of the [^14^C]sucrose *R*_Tissue_ value. Blood-to-brain unidirectional rate constants (*K*_in_) were determined by single-time uptake analysis (*K*_in_ = [^14^C]sucrose-corrected *R*_Tissue_ values/perfusion time) after 10 min of perfusion (Williams *et al.*, 1996). [^14^C]Sucrose is a baseline marker. In brain samples, it provided a measurement of vascular space. Any deviation from the norm indicated loss of BBB integrity. In the choroid plexus, [^14^C]sucrose provides measures of the vascular space and extracellular spaces formed between the choroidal capillary endothelium and epithelium.

### Octanol/saline partition coefficient

An octanol/saline partition coefficient (pH 7.4) was determined for [^3^H]AM 580 (6.0 nm), using previously described methods (Sanderson *et al.*, 2008).

### Data analysis

Data were analysed with Student’s *t-*test or one-way anova, followed by Tukey’s test with sigma stat software (SPSS Software, Birmingham, UK). Comparisons were made between appropriate groups, and differences were considered to be statistically significant at a *P*-level of 0.05. Results are means ± standard errors (SEs), and *P*-values are provided as summary statistics.

## Results

**Fig. 1:**
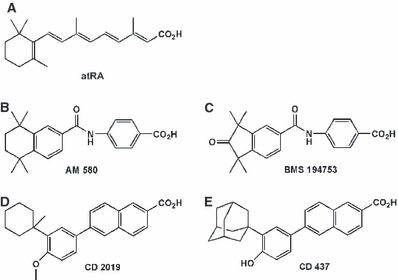
Structure of the retinoid agonists. (A) atRA, a pan-agonist of all the RARs. (B) AM 580, an RARα agonist. (C) BMS 194753, an RARα agonist. (D) CD 2019, an RARβ agonist. (E) CD 437, an RARγ agonist.

**Fig. 2:**
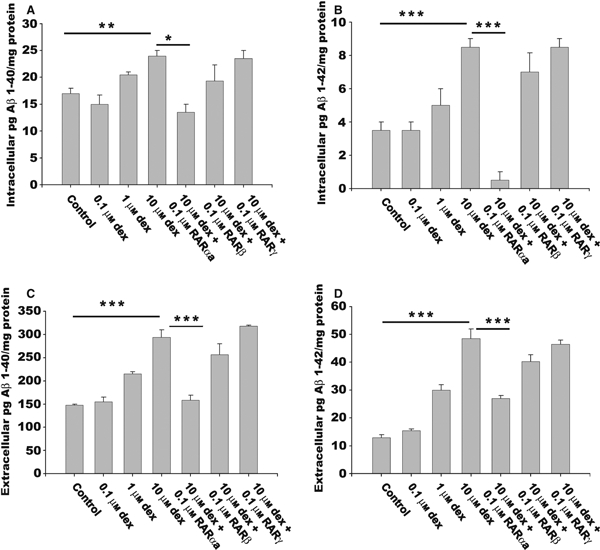
RARα signalling prevents both intracellular and extracellular Aβ accumulation. Cortical neurons were cultured in the presence of either 0.1, 1 or 10 μm dexamethasone (Dex) with or without 0.1 μm retinoids for 3 days; they were then assayed for intracellular and extracellular Aβ1–40 and Aβ1–42 by ELISA. (A) Intracellular Aβ1–40. (B) Intracellular Aβ1–42. (C) Extracellular Aβ1–40. (D) Extracellular Aβ1–42. With increasing amounts of dexamethasone, there were increases in both extracellular and intracellular Aβ1–40 and Aβ1–42 accumulation as compared with control cultures. In the presence of RARα agonist and 10 μm dexamethasone, there were decreases in the amounts of both extracellular and intracellular Aβ1–40 and Aβ1–42 as compared with the 10 μm dexamethasone-treated cultures. Student’s *t*-test for dose–response study and one-way anova followed by Tukey’s test for comparison between retinoid treatments. Results are mean ± SE (*n* = 3; *P < 0.05, **P < 0.005, ***P < 0.001).

It has previously been shown that the glucocorticoid dexamethasone induces Aβ production in mouse neuronal N2A cells (Green *et al.*, 2006) by altering APP processing. Therefore, to investigate the potential role of retinoid signalling in Aβ production, we treated mouse embryonic day 15 cortical neurons with dexamethasone and retinoid agonists (Fig. 1). Cortical neurons were cultured in serum-free medium, and after 7 days, 0.1, 1 or 10 μm dexamethasone was added to the cultures; 3 days later, both intracellular and extracellular Aβ was measured by sandwich ELISA. With increasing amounts of dexamethasone, there were increases in intracellular and extracellular Aβ1-40 as compared with vehicle-treated cultures (Fig. 2A and C; intracellular Aβ1–40, Student’s *t*-test, *t*_4_ = −7.23, *P* = 0.002, vehicle vs. 10 μm dexamethasone; extracellular Aβ1–40, Student’s *t*-test, *t*_4_ = −12.00, *P* < 0.001, vehicle vs. 10 μm dexamethasone) and increases in intracellular and extracellular Aβ1–42 as compared with vehicle-treated cultures (Fig. 2B and D; intracellular Aβ1–42, Student’s *t*-test, *t*_4_ = −10.60, *P* < 0.001, vehicle vs. 10 μm dexamethasone; extracellular Aβ1–42, Student’s *t*-test, *t*_4_ = −16.28, *P* < 0.001, vehicle vs. 10 μm dexamethasone). In order to establish whether RAR agonists could prevent this Aβ production caused by dexamethasone, neurons were cultured as above, and on day 7, 10 μm dexamethasone and 0.1 μm RARα, RARβ or RARγ agonist was added. After 3 days, sandwich ELISAs to detect Aβ1–40 and Aβ1–42 were carried out. In the presence of the RARαa agonist, there were significant decreases in the amounts of intracellular and extracellular Aβ1–40 as compared with 10 μm dexamethasone-treated cultures (Fig. 2A and C; intracellular Aβ1–40, one-way anova, *P* = 0.008, followed by Tukey’s test, *F*_3,8_ = 8.28, *P* = 0.008; extracellular Aβ1–40, one-way anova, *P* < 0.001, followed by Tukey’s test, *F*_3,8_ = 25.81, *P* = 0.006) and of intracellular and extracellular Aβ1–42 as compared with 10 μm dexamethasone-treated cultures (Fig. 2B and D; intracellular Aβ1–42, one-way anova, *P* < 0.001, followed by Tukey’s test, *F*_3,8_ = 32.24, *P* < 0.001; extracellular Aβ1–42, one-way anova, *P* < 0.001, followed by Tukey’s test, *F*_3,8_ = 35.84, *P* < 0.001). In contrast RARβ and RARγ agonists had little or no effect on either intracellular or extracellular levels of Aβ1-40 and Aβ1-42 as compared with dexamethasone-treated cultures (Fig. 2A–D; one-way anova followed by Tukey’s test as above, all *P* > 0.05).

**Fig. 3:**
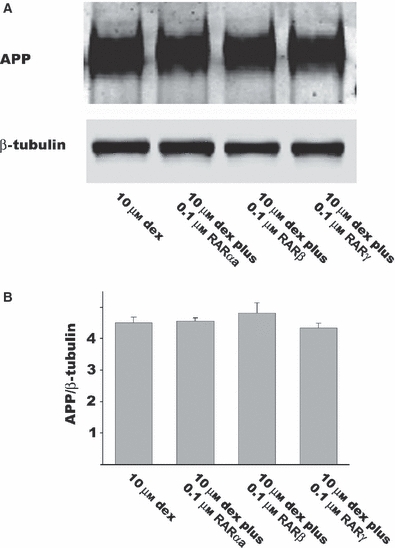
Effect of retinoids on APP expression. Cortical neurons were cultured in serum-free medium for 7 days, and then treated with 10 μm dexamethasone with or without 0.1 μm retinoid agonists for 3 days. (A) Western blot of total APP. (B) Quantification of total APP. There was no significant change in total levels of APP in the presence of any of the retinoid agonists as compared with dexamethasone-treated cultures. One-way anova. Results are mean ± SE (*n* = 3).

Previous work has shown that dexamethasone increases Aβ production by increasing levels of both APP and BACE1 (Green *et al.*, 2006). Therefore, we next investigated whether RARα signalling was affecting total APP levels and/or those of the enzymes involved in its processing. On western blotting, there was no significant increase in total APP expression in dexamethasone-treated cultures as compared with dexamethasone/retinoid agonist-treated cultures (Fig. 3A and B; one-way anova, *P* = 0.76, *F*_3,8_ = 0.392, all *P* > 0.05).

**Fig. 4:**
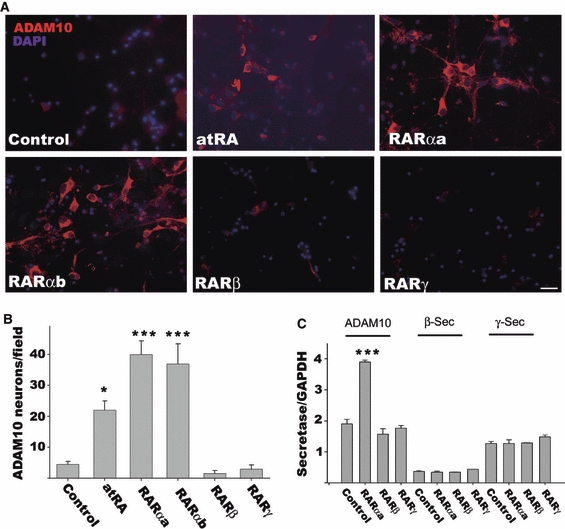
Effect of retinoids on secretases involved in APP processing. Cortical neurons were cultured in serum-free medium for 7 days, and then treated with 0.1 μm retinoid agonist for 3 days. (A) Immunohistochemistry of ADAM10 expression. (B) Quantification of ADAM10 expression. (C) Lightcycler RT-PCR of ADAM10, β-secretase and γ-secretase; RARα signalling increased ADAM10 expression, whereas none of the retinoids altered the levels of β-secretase or γ-secretase. Student’s *t*-test. Results are mean ± SE (*n* = 3; *P < 0.05, ***P < 0.001). Scale bar – 50 μm.

By immunohistochemistry, there was an increase in the number of cortical neurons expressing the α-secretase ADAM10 in the presence of 0.1 μm atRA as compared with vehicle-treated cultures (Fig. 4A and B; one-way anova, *P* < 0.001, followed by Tukey’s test, *F*_5,12_ = 75.10, *P* = 0.016). In neurons cultured in the presence of either 0.1 μm RARαa or 0.1 μm RARαb, there was also a significant increase in ADAM10 expression as compared with vehicle-treated cultures (Fig. 4A and B; one-way anova, followed by Tukey’s test as above, RARαa and RARαb, *P* < 0.001), whereas neither RARβ nor RARγ agonists affected ADAM10 expression as compared with vehicle-treated cultures (Fig. 4A and B; one-way anova, followed by Tukey’s test as above, *P* > 0.05). These data were confirmed by light cycler RT-PCR (Fig. 4C); the RARαa agonist increased ADAM10 expression as compared with vehicle-treated cultures (Fig. 4C; one-way anova, *P* < 0.001, followed by Tukey’s test, *F*_3,8_ = 278, *P* < 0.001), whereas neither RARβ or RARγ agonists affected ADAM10 expression as compared with vehicle-treated cultures (Fig. 4C; one-way anova, followed by Tukey’s test as above, all *P* > 0.05). None of the retinoids affected β-secretase expression as compared with vehicle-treated cultures (Fig. 4C; one-way anova, *P* = 0.005, *F*_3,8_ = 5.20, all *P* > 0.05) or γ-secretase expression as compared with vehicle-treated cultures (Fig. 4C; one-way anova, *P* = 0.265, *F*_3,8_ = 1.38, all *P* > 0.05). These data suggest that RARα signalling processes the APP into the non-amyloidic pathway by upregulating ADAM10 expression and that none of the agonists affects the amyloidic pathway.

**Fig. 5:**
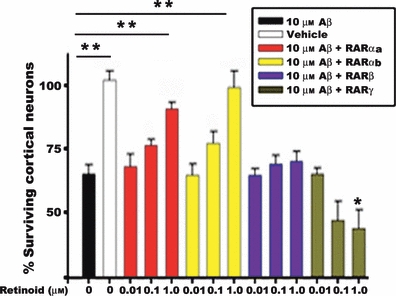
Effect of retinoids on Aβ-mediated neuronal cell death in cortical neuronal cultures. Cortical neurons were cultured in serum-free medium for 7 days, and then treated with 10 μm Aβ1-42 alone or with either 0.1, 1 or 1 μm retinoid agonist for 3 days. Cell survival was measured by an MTT assay. In the presence of 10 μm Aβ1–42, there was a significant amount of neuronal cell death as compared with control cultures; this could be attenuated by RARα signalling, but not by RARβ or RARγ signalling. Student’s *t*-test. Results are mean ± SE (*n* = 3; *P < 0.05, **P < 0.005).

Given that the α-secretase products are neuroprotective (Allinson *et al.*, 2003), we investigated whether RARα signalling could prevent Aβ-induced neuronal cell death. Cortical neurons were cultured in serum-free medium for 7 days, and then in the presence of 10 μm human Aβ1–42 alone or in combination with either 0.01, 0.1 or 1 μm retinoid agonist. Three days later, neuronal cell death was assessed with an MTT assay. In the presence of Aβ1–42, there was a significant amount of neuronal cell death as compared with the control cultures (Fig. 5; Student’s *t*-test, *t*_4_ = −3.47, *P* = 0.003). This cell death could be prevented by the RARα agonists, where there was increasing neuronal cell survival with increasing dose of the agonist (Fig. 5; Student’s *t*-test, *t*_4_ = −3.38, *P* = 0.004 for 1 μm RARαa; Student’s *t*-test, *t*_4_ = −3.47, *P* *=*0.003 for 1 μm RARαb). With increasing dose of the RARβ agonist, there was little or no effect on cell survival (Fig. 5; Student’s *t*-test, *t*_4_ = −0.37, *P* = 0.071 for 1 μm RARβ), and only the highest concentration of RARγ used had an effect that contributed to cell death (Fig. 5; Student’s *t*-test, *t*_4_ = 3.02, *P* = 0.008 for 1 μm RARγ). Similar data were obtained with the use of propidium iodide and Hoescht (data not shown).

**Fig. 6:**
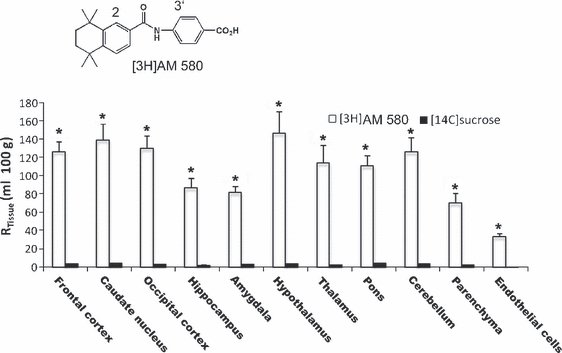
Retinoids can cross the BBB. [^3^H]AM 580 was prepared with two tritium labels, one each in the 2 and 3′ positions, as shown. The graph shows [^3^H]AM 580 and [^14^C]sucrose (vascular space marker) detected in brain regions and parenchyma (supernatant) and capillary endothelial cells (pellet) after capillary depletion analysis of the whole brain homogenate relative to that in the plasma (*R*_Tissue_; mL/100 g). One-way anova on ranks, *P* < 0.001, followed by Tukey’s test, *P* <0.05 for both brain region samples and capillary depletion samples. Results are mean ± SE (*n* = 3–5; *P < 0.05 for [^3^H]AM 580 as compared with [^14^C]sucrose).

To assess whether retinoid agonists could be used to affect Aβ load *in vivo*, we investigated whether retinoids could pass through the BBB by using [^3^H]AM 580 (Fig. 6) and an *in situ* brain perfusion technique in anaesthetized mice. All [^14^C]sucrose values in the brain regions, capillary depletion and choroid plexus samples were as expected. There was a significant difference between the values obtained for [^3^H]AM 580 and [^14^C]sucrose in all brain region and capillary depletion samples (Fig. 6; one-way anova on ranks, *P* *<* 0.001, followed by Tukey’s test, *P* *<* 0.05, for both brain region samples and capillary depletion samples; one degree of freedom). The amount of [^3^H]AM 580 (78.7 ± 6.7%, *n* = 5, corrected for vascular space) in the amygdala was approximately 32 times greater than the amount of [^14^C]sucrose, and was significantly lower (one-way repeated measures anova, *P* *<* 0.001, *F*_8,88_ = 6.848, followed by Tukey’s test) than that in the hypothalamus (*P* *<* 0.001), caudate nucleus (*P* *=* 0.001), occipital cortex (*P* *=* 0.006), frontal cortex (*P* *=* 0.016) and cerebellum (*P* *=* 0.016) (Fig. 6). The *K*_in_ value for [^3^H]AM 580 ranged from 78.7 ± 6.7 μL/min per g in the amygdala to 142.7 ± 23.7 μL/min per g in the hypothalamus. [^3^H]AM 580 was also detected in the CSF at higher levels than [^14^C]sucrose (3.6 ± 0.67%, [^14^C]sucrose values removed; *n* = 3). [^3^H]AM 580 was found at very high levels in the choroid plexus, *R*_Tissue_ values reaching 993.1 ± 78.0% (*n* = 5; [^14^C]sucrose values corrected). The octanol/saline partition coefficient measured for [^3^H]AM 580 was 207.8 ± 38.0% (*n* = 3).

**Fig. 7:**
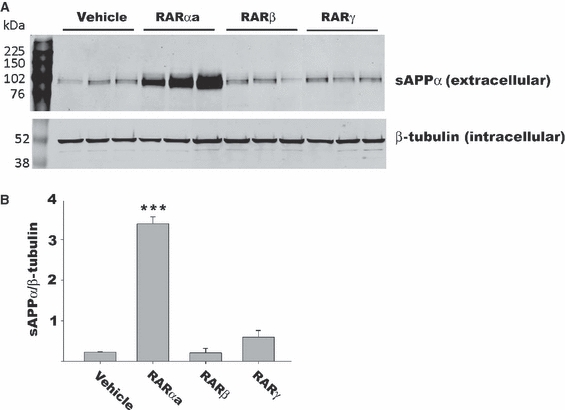
RARα signalling increases sAPPα production in Tg2576 mice. Mice were injected intraperitoneally with 1 mg/kg retinoids from 3 to 7 months of age every 3 days. (A) Western blot of sAPPα production in cortices. (B) Quantification of sAPPα production in cortices. There was a significant increase in sAPPα production in the RARα agonist-treated mice as compared with the vehicle-treated ones, whereas the other RAR agonists had no effect on its production. One-way anova, followed by Tukey’s test. Results are mean ± SE (*n* = 3; *P < 0.05).

Having established that retinoids can cross the BBB, we investigated whether any of the retinoid agonists had an effect on the Aβ load in Tg2576 mice, which overexpress the Swedish mutation of APP, leading to increased Aβ levels (Hsiao *et al.*, 1996). They were injected intraperitoneally three times a week with 1 mg/kg of either vehicle, RARαa, RARβ or RARγ agonist from 3 to 7 months of age, or fed 3.6 mg/kg of either RARαa or RARαb. We first investigated whether there was an increase in cortical extracellular sAPPα protein, which is a product of α-secretase processing (Thinakaran & Koo, 2008) by RARα signalling. By western blotting, there was a significant increase in the levels of this protein in the RARα agonist-treated mice as compared with the vehicle-treated ones (Fig. 7A and B; one-way anova, *P* < 0.01, followed by Tukey’s test, *F*_3,8_ = 129, *P* < 0.001), whereas the RARβ or RARγ agonists had no effect on this protein as compared with the vehicle-treated mice (Fig. 7A and B; one-way anova, followed by Tukey’s test as above, *P* > 0.05).

**Fig. 8:**
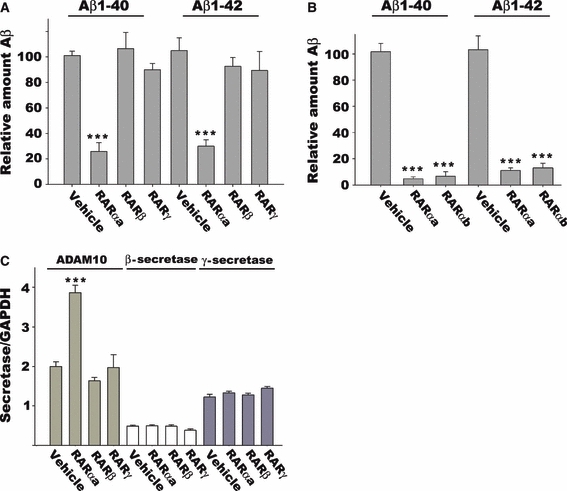
RARα signalling prevents Aβ accumulation in Tg2576 mice by upregulating ADAM10 expression. Mice were injected intraperitoneally with 1 mg/kg retinoids from 3 to 7 months of age every 3 days, or they were fed RARα agonists, 3.6 mg/kg, from 3 to 7 months of age. Aβ1–40 and Aβ1–42 levels were measured by ELISA. (A) Aβ1–40 and Aβ1–42 levels in intraperitoneal RAR agonist-treated mice. (B) Aβ1–40 and Aβ1–42 levels in RARα agonist-fed mice. (C) Expression of secretases in intraperitoneal RAR agonist-treated mice. There were significant decreases in Aβ1–40 and Aβ1–42 levels in the RARα agonist-treated mice as compared with the vehicle-treated ones, whereas the other RAR agonists did not alter Aβ load. In only the RARα agonist-treated mice was there a significant increase in ADAM10 expression as compared with the vehicle-treated ones; none of the retinoid agonists affected β-secretase or γ-secretase. One-way anova, followed by Tukey’s test. Results are mean ± SE (*n* = 3–5; ***P < 0.001).

To confirm that the increase in sAPPα levels caused by RARα signalling correlated with a decrease in Aβ levels, we carried out ELISAs for Aβ1–40 and Aβ1–42. In the cortices, there was a significant decrease in the amounts of both Aβ1–40 and Aβ1–42 in the RARα agonist-treated mice given agonist intraperitoneally as compared with the vehicle-treated ones (Fig. 8A; for Aβ1–40 levels, one-way anova, *P* *<* 0.001, followed by Tukey’s test, *F*_3,8_ = 68.40, *P* < 0.001; for Aβ1–42 levels, one-way anova, *P* *<* 0.001, followed by Tukey’s test, *F*_3,8_ = 33.90, *P* < 0.001) or orally as compared with the vehicle-treated ones (Fig. 8B; for Aβ1–40 levels, one-way anova, *P* < 0.001, followed by Tukey’s test, *F*_2,12_ = 810, all *P* < 0.001; for Aβ1–42 levels, one-way anova, *P* < 0.001, followed by Tukey’s test, *F*_2,12_ = 352, all *P* < 0.001). There was no significant difference between Aβ1-40 and Aβ1–42 levels in the RARβ-treated or RARγ-treated Tg2576 mice as compared with the vehicle-treated ones (Fig. 8A; one-way anova, followed by Tukey’s test as above for intraperitoneal treatment, all *P* > 0.05).

We finally investigated whether *in vivo* levels of ADAM10, β-secretase and/or γ-secretase were altered by the retinoids. By light cycler RT-PCR, there was a significant increase in ADAM10 expression in RARαa agonist-treated mice as compared with vehicle-treated ones (Fig. 8C; one-way anova, *P* < 0.001, followed by Tukey’s test, *F*_3,8_ = 60.06, *P* *<*0.001), whereas RARβ or RARγ agonists had no effect on its expression (Fig. 8C; one-way anova followed by Tukey’s test as above, *P* > 0.05). None of the retinoid agonists affected β-secretase (Fig. 8C; one-way anova, *P* = 0.11, *F*_3,8_ = 2.32, all *P* > 0.05) or γ-secretase (Fig. 8C; one-way anova, *P* = 0.06, *F*_3,8_ = 3.00, all *P* > 0.05) expression as compared with the vehicle-treated mice. These data suggest that the increase in α-secretase products is primarily attributable to an increase in ADAM10 expression, and this leads to APP being processed into the non-amyloidic pathway, resulting in decreased Aβ1–40 and Aβ1–42 production in the Tg2576 mice.

## Discussion

Previous work has shown that the loss of RA signalling in the rat caused by dietary deficiency of vitamin A can cause Aβ deposition (Corcoran *et al.*, 2004) and alterations in enzymes involved in APP processing (Husson *et al.*, 2006), and that there is a loss of RARα expression in AD human pathology samples (Corcoran *et al.*, 2004). Recent data have shown that atRA, a pan-agonist of the RARs, given intraperitoneally to a mouse model of AD can reduce Aβ levels (Ding *et al.*, 2008). By using the glucocorticoid dexamethasone, we have shown here that the production of Aβ is increased in embryonic mouse cortical neurons, and similar data have been obtained in N2A and PC12 cells (Snyder *et al.*, 2005; Green *et al.*, 2006), further highlighting the association between stress hormones and AD (Dong & Csernansky, 2009). The increase in Aβ can be attenuated by RA signalling, and we have shown here that it is RARα as opposed to RARβ and RARγ signalling that carries out this role.

One mechanism through which RARα signalling acts is by modulating the processing of APP into the non-amyloidic rather than the amyloidic pathway, without affecting the overall levels of APP. In the non-amyloidic pathway, APP can be cleaved by α-secretases into a soluble neuroprotective fragment (Allinson *et al.*, 2003), and one of these, ADAM10, has been shown to be regulated by RA (Prinzen *et al.*, 2005; Koryakina *et al.*, 2009; Tippmann *et al.*, 2009), and this may be a direct action, as the promoter of this gene contains an RA response element (Prinzen *et al.*, 2005). ADAM10 cleaves the APP located on the cell surface, thus preventing the build-up of extracellular Aβ (Allinson *et al.*, 2003). Although RAR needs to heterodimerize with RXR in order to activate gene transcription, RXR agonists alone do not induce ADAM10 expression, suggesting that RAR agonists are essential for its expression (Tippmann *et al.*, 2009). Here we have shown both *in vitro* and *in vivo*, that RARα signalling increases ADAM10 expression, whereas neither RARβ nor RARγ activation have little or no effect on its expression. In the amyloidic pathway, APP is cleaved by β-secretase and γ-secretase (Thinakaran & Koo, 2008), which we have shown are not regulated by any of the retinoid agonists.

In addition to RARα signalling decreasing extracellular Aβ, we have demonstrated here that it also decreases intracellular Aβ. It has been shown that intraneuronal Aβ plays a role in cognitive dysfunction (Billings *et al.*, 2005), and that intraneuronal Aβ leads to neuronal cell death (Kienlen-Campard *et al.*, 2002). The source of this intracellular Aβ may be secreted Aβ that is taken back into the cell, or non-secreted Aβ (LaFerla *et al.*, 2007). Retinoids have already been shown to be neuroprotective (Wuarin & Sidell, 1991; Ahlemeyer *et al.*, 2001; Corcoran *et al.*, 2002, 2004; Hagglund *et al.*, 2006), but the mechanism by which they achieve this has not yet been shown. Given that RARα agonists increase APP processing into the non-amyloidic pathway and that this gives rise to a neuroprotective fragment, this may help to prevent Aβ toxicity.

We have shown that, *in vivo*, AM 580 can cross the BBB. [^3^H]AM 580 can be detected in the brain parenchyma and in the cerebral capillary endothelial cells, indicating detectable BBB passage. It can be estimated that 2.5 ± 0.2% of the injected dose of [^3^H]AM 580 reaches 1 g of murine brain in 10 min. This relatively high distribution to the brain is likely to be partly associated with the considerable lipophilicity of [^3^H]AM 580 as measured by the octanol/saline partition coefficient.

In addition, there was significant variation in the regional brain distribution of [^3^H]AM 580 as shown by differences in *K*_in_ values. This may be related to the differential localization of transporters for [^3^H]AM 580 at the BBB. Interestingly, STRA6 is a multitransmembrane domain protein that has been demonstrated to be involved in retinol absorption in bovine retinal pigment epithelial cells (Bouillet *et al.*, 1997; Kawaguchi *et al.*, 2007). STRA6-expressing capillaries are found specifically in the striatum, preoptic zone, medial cerebellar nuclei and ventral cochlea nuclei, and STRA6 is also densely expressed in the choroid plexus (Bouillet *et al.*, 1997). Thus, it is of interest that a significant amount of [^3^H]AM 580 was associated with the murine choroid plexus samples; however, it has not been determined whether STRA6 can bind the retinoid agonists. Our study shows that [^3^H]AM 580 is able to cross the blood–CSF barrier and be detected in the CSF. It is important to note that CSF concentrations are considered to reflect unbound drug concentrations in brain interstitial fluid (Liu *et al.*, 2009).

We have used two different structural classes of RARα agonists, which give the same results *in vivo*, strongly pointing to this being an RARα action. The administration of the RARα agonist *in vivo*, as *in vitro*, leads to an upregulation in ADAM10 expression and a reduction in Aβ production. We have shown that, by giving the retinoid agonists to the Tg2576 mice either intraperitoneally or orally for 3 months from 3 to 7 months of age, a period when there is a dramatic increase in Aβ production (Kawarabayashi *et al.*, 2001), this is prevented. An orally given drug would be an attractive therapeutic agent for AD, and we note that there was a greater decrease in Aβ production with the oral than with the intraperitoneal route. On the one hand, this may be because the oral dosing was continuous, such that the drug was not cleared; on the other hand, it may be that the oral dose was simply higher than the intraperitoneal dose.

In summary, we have shown here that RARα agonists have multiple effects on pathways in AD. They process APP into the non-amyloidic pathway, they decrease intracellular Aβ production, and they are neuroprotective. Given the diversity of these roles, we suggest that RARα agonists have therapeutic potential for the treatment of AD, and have clear advantages over current drugs, such as cholinesterase inhibitors, which may target only one aspect of the disease.

## Abbreviations

AD: Alzheimer’s disease
ADAM: a disintegrin and metalloprotease
APP: amyloid precursor protein
atRA: all-trans retinoic acid
Aβ: amyloid-β
BACE1: β-site of amyloid precursor protein-cleaving enzyme
BBB: blood–brain barrier
CSF: cerebrospinal fluid
DAPI: 4′,6-diamidino-2-phenylindole dihydrochloride
ELISA: enzyme-linked immunosorbent assay, GAPDH, glyceraldehyde-3-phosphate dehydrogenase
MTT: 3-(4,5-dimethylthiazol-2-yl)-2,5-diphenyltetrazolium bromide
PBS: phosphate-buffered saline
PCR: real-time polymerase chain reaction
RA: retinoic acid
RAR: retinoic acid receptor
RT: reverse transcription
RXR: retinoid X receptor
sAPP: secretory APP
SE: standard error
